# Machine learning-aided atomic structure identification of interfacial ionic hydrates from AFM images

**DOI:** 10.1093/nsr/nwac282

**Published:** 2022-12-14

**Authors:** Binze Tang, Yizhi Song, Mian Qin, Ye Tian, Zhen Wei Wu, Ying Jiang, Duanyun Cao, Limei Xu

**Affiliations:** International Center for Quantum Materials, Peking University, Beijing100871, China; School of Physics, Peking University, Beijing100871, China; International Center for Quantum Materials, Peking University, Beijing100871, China; School of Physics, Peking University, Beijing100871, China; School of Physics, Peking University, Beijing100871, China; International Center for Quantum Materials, Peking University, Beijing100871, China; School of Physics, Peking University, Beijing100871, China; Institute of Nonequilibrium Systems, School of Systems Science, Beijing Normal University, Beijing 100875, China; International Center for Quantum Materials, Peking University, Beijing100871, China; School of Physics, Peking University, Beijing100871, China; Collaborative Innovation Center of Quantum Matter, Beijing100871, China; CAS Center for Excellence in Topological Quantum Computation, University of Chinese Academy of Sciences, Beijing 100049, China; Interdisciplinary Institute of Light-Element Quantum Materials and Research Center for Light-Element Advanced Materials, Peking University, Beijing100871, China; Beijing Key Laboratory of Environmental Science and Engineering, School of Materials Science and Engineering, Beijing Institute of Technology, Beijing100081, China; Beijing Institute of Technology Chongqing Innovation Center, Chongqing401120, China; International Center for Quantum Materials, Peking University, Beijing100871, China; School of Physics, Peking University, Beijing100871, China; Collaborative Innovation Center of Quantum Matter, Beijing100871, China; Interdisciplinary Institute of Light-Element Quantum Materials and Research Center for Light-Element Advanced Materials, Peking University, Beijing100871, China

**Keywords:** machine learning, transfer learning, atomic force microscopy, atomic scale structure identification, interfacial ion hydrates

## Abstract

Relevant to broad applied fields and natural processes, interfacial ionic hydrates have been widely studied by using ultrahigh-resolution atomic force microscopy (AFM). However, the complex relationship between the AFM signal and the investigated system makes it difficult to determine the atomic structure of such a complex system from AFM images alone. Using machine learning, we achieved precise identification of the atomic structures of interfacial water/ionic hydrates based on AFM images, including the position of each atom and the orientations of water molecules. Furthermore, it was found that structure prediction of ionic hydrates can be achieved cost-effectively by transfer learning using neural network trained with easily available interfacial water data. Thus, this work provides an efficient and economical methodology that not only opens up avenues to determine atomic structures of more complex systems from AFM images, but may also help to interpret other scientific studies involving sophisticated experimental results.

## Teaser

With the aid of machine learning, a new methodology is proposed to automatically and cost-effectively interpret atomic force microscopy images of complex systems like interfacial water/ ionic hydrates.

## INTRODUCTION

Interfacial ionic hydrates are ubiquitous in nature and are closely related to a variety of essential issues in applied fields and natural processes, from electrocatalytic processes [1–4], seawater desalination [5–8], to biological ion channels [9,10] and chemical reactions [11,12]. The structural information of ionic hydrates at the atomic level is crucial for elucidating various extraordinary physical and chemical properties of ionic hydrate/solid interfaces. In recent years, qPlus-based non-contact atomic force microscopy (AFM) with a CO-decorated tip [13], capable of directly imaging water nanoclusters with submolecular resolution [14–18], has emerged as the most promising candidate for characterizing interfacial water network structures and dynamics at the atomic level [19]. However, in sharp contrast to the oxygen skeleton, which can be clearly reflected, identifying hydrogen atoms from AFM images is a challenge because they are barely visible [20]. Therefore, accurate structure identification, by combining density functional theory (DFT)-based stability calculations and probe-particle-method-based AFM simulations [21,22], typically requires many trial-and-error processes to exclude a large number of possible structural models with different OH orientations.

Recently, machine learning (ML) has been used in various scientific and technological studies [23–28], such as structure identification [29–32], structure discovery [29,33,34] and electrostatic discovery [35] related to microscopy imaging. Especially for AFM imaging, a convolution neural network (CNN) has been applied by Alldritt *et al.* to resolve the configuration of organic molecules [36]. In their study, the feeding data for ML were the simulated AFM images generated based on DFT-optimized structures from the pre-existing databases. However, such an approach is not suitable for interfacial ionic hydration systems, since there is no ready database including ionic hydrate structures, and it is costly and impractical to acquire such large numbers of structures based on DFT calculations. In addition, this ML method compresses the 3D structure into 2D convolution and has no skip connections in neural network (NN) architecture, which may lead to the loss of structural information during the NN processing process of AFM images. Moreover, signals of hydrogen atoms are weaker than those of other atoms (such as oxygen), which further increases the difficulty of identifying H. Therefore, it requires a cost-effective way to obtain input data, as well as a good structure representation and an effective NN architecture to distinguish H from other atoms.

Generally, the generation of input data consists of two key steps: structure acquisition and AFM image simulation. Structure acquisition requires sufficient phase space sampling of the target system to effectively introduce structural physics-induced biases into the NN [24]. Obviously, the more accurate and richer the input structure, the higher the prediction accuracy. The DFT-based method has high accuracy, but cannot sample enough structures for training due to the great demands for computational resources. As an alternative, the classical molecular dynamic (MD) simulations can provide many plausible structures economically and quickly [37]. At the same time, its lack of calculated energy accuracy has a minor impact on structure prediction and is therefore negligible in structure acquisition for ML.

In terms of AFM simulations, calculations of the potential surface distribution of the system used for AFM simulations must be accurate enough to agree well with the experimental results. Without the need for DFT calculations to obtain the system electrostatic potential, the classical point-charge model incorporating Lennard–Jones (L–J) interactions [21,22] can be used to cost-effectively perform AFM simulations of interfacial water. However, when ions are added, this point-charge method no longer accurately provides the potential surface for the ionic hydrate system, so DFT calculations become crucial. Fortunately, transfer learning [38] can be used to transfer knowledge from interfacial water systems to the interfacial ionic hydrate systems because their hydrogen-bond (H-bond) networks share similar characteristics. Such usage can nicely reduce the need for interfacial ionic hydrate data, thus making the training of NNs for ionic hydrates more economical.

Therefore, taking Na^+^ hydrates, one of the most abundant alkali-metal ions in nature, as an example, we designed an ML method with transfer learning to economically determine the structure of interfacial ionic hydrates from the AFM images. A NN was first trained on a large number of AFM images of interfacial water structures simulated via classical MD and then retrained via transfer learning on AFM images of Na^+^ hydrates simulated based on DFT-computed electrostatic potentials. Strikingly, using only thousands of simulated data of Na^+^ hydrates, the prediction accuracy of the retrained NN can reach 95% for both sodium and oxygen, and 85% even for hydrogen. From the designed structure representations predicted by NNs, the positions of each atom and the orientations of water molecules can be easily identified, which has not been achieved by any ML method to date. The accuracy and efficiency of this prediction far exceed the trial-and-error process of human experts. Furthermore, this economical ML method is also a fairly general workflow for structure prediction from AFM images and can be extended from interfacial ionic hydrate systems to other complex systems, such as the surface of bulk ice, and even to identify information from the results of other spectroscopic experiments [34,39–41].

## RESULTS

**Figure 1.**
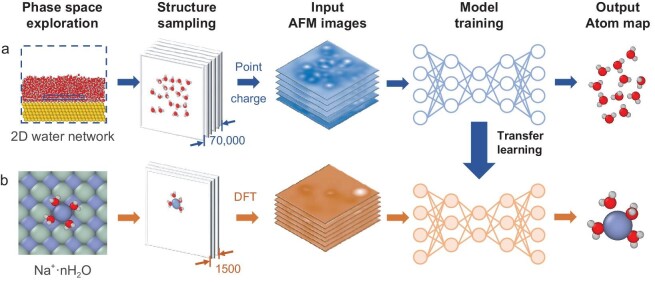
Schematic illustration of the overall framework of the training processes. Training processes (a) and (b) are performed sequentially. First, 70 000 2D interfacial water network structures are sampled, based on which AFM images are simulated with point-charge electrostatic potentials as the input for the training process in (a). After training, simulated AFM images of 1500 Na^+^ hydrate structures are generated with DFT-calculated electrostatic potentials. These data are the input into the transfer-learning process in (b). The output of the NN is a 2D representation of the molecular structure which looks similar to the atom map shown here.

As illustrated in Fig. 1, the workflow consists of preliminary training and retraining for structure prediction for interfacial water (Fig. 1a) and interfacial Na^+^ hydrates (Fig. 1b), respectively. During preliminary training, the NN, which is a standard 3D U-Net model [42,43] (see ‘Methods’ section), is fed with sufficient interfacial water data to learn to resolve the structure information of H-bond networks from AFM images. Then, via transfer learning, the NN is retrained with a small amount of expensive interfacial Na^+^ hydrates data to achieve efficient structure identification of Na^+^ hydrates economically. In both cases, the implemented learning schemes include data preparation, data labeling, NN training and performance evaluation. Data preparation, including phase space exploration based on classical MD simulations, structure sampling, AFM images simulation and data processing, is the key to transfer learning and is described in more detail below. For the identification of hydrogen atoms, the designed structure representation (data label) and the corresponding evaluating criteria will also be stated later.

### Data preparation for preliminary training

A large number of simulated AFM images of well-sampled different structures from the interfacial water layer on the Au(111) substrate were used in the preliminary NN training to ensure the robustness of the NN. Considering the large density variation in different layers [44], the structure sampling in the sampled phase space was performed by sliding a detection window of $2.5 \times 2.5 \times 0.3\ {\rm {nm}}^3$ along the direction parallel and perpendicular to the substrate (Fig. 1a, the first panel). We note that the detection window is large enough to cover various interfacial water structures in the XY plane and ensure that AFM can only probe one layer along the Z direction (see Supplementary Information (SI) Section 4 for details). Up to 80 000 2D water structures of different temperatures and densities were selected for AFM simulations, of which 70 000 and 10 000 were randomly selected for training and for validation, respectively. For each structure, 10 images were randomly selected from a set of simulated AFM images with a tip-sample distance (defined in the ‘Methods’ section) of 1.14 to 1.34 nm (10-pm intervals) and stacked as the input data for NN training. Data augmentation including cutout, noise and pixel shift was implemented before NN training to prevent overfitting (see SI Section 5 for details).

### The NN output and performance assessment for structure prediction

**Figure 2.**
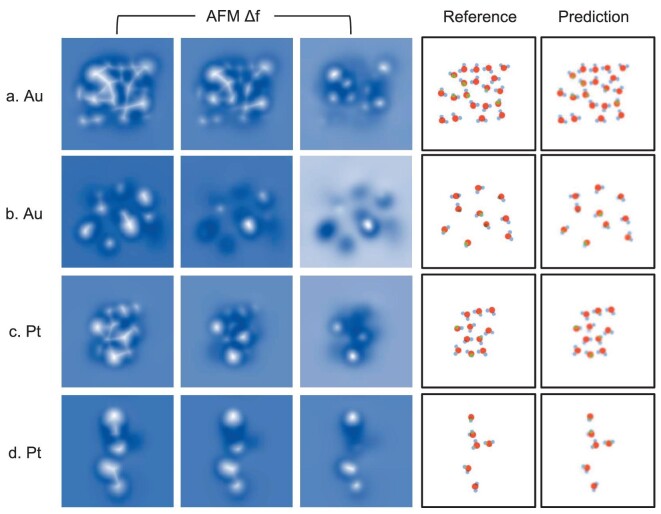
Examples of network prediction based on interfacial water simulation data. (a) A dense water layer and (b) a dilute water layer on the Au surface selected from the validation data set. (c) and (d) are the test data for dense and dilute water layers on the Pt surface to examine network transferability, respectively. Columns 1–3 are simulated AFM images (input data) with increasing tip-sample distances. Column 4 is the a-vdW sphere representation of the structure (label) and Column 5 is the prediction from the network. The red dots represent oxygen atoms, and the light blue, green and brown dots represent hydrogen atoms at the same height as above and below the oxygen atoms, respectively.

An ideal output of structure prediction should include chemical identity and positions of atoms. Considering that NN cannot effectively predict individual coordinate values and specific atom types [36], we designed an intuitive representation to encode molecular structures, namely advanced vdW (a-vdW) spheres. In this representation, atoms are represented by van der Waals radius ($\sigma $) and dispersion energy ($\epsilon $) of the L–J interaction, as well as the charge *q*. Thus, a colored atom map (Fig. 2, right two panels) can be obtained as the NN output (see SI Section 6 for details), from which atomic identities and positions can be easily distinguished. We stress that this representation also contains some 3D structure information, because atomic circles are stacked sequentially with increasing height, so that the uppermost of the overlapping atomic circles represents the highest atom.

The per-pixel mean square error (MSE) calculated between the reference image and the predicted image was used as the loss function of NN training to thoroughly evaluate the predictions for each structure. Moreover, considering that the goal of NN is object detection, we designed an algorithm to locate the positions of all atoms and further calculated the prediction accuracy of each atomic species for intuitively evaluating the NN performance (see SI Section 16 for details). For each atomic species, the prediction accuracy was calculated based on all predictions for it in the whole data set, rather than averaging its prediction accuracy for each structure. This is because the small number of atoms in a particular structure results in discontinuity of the prediction accuracy and further leads to inaccurate statistical results. The error bar for the prediction accuracy was calculated from the data at the top and bottom 20% of the MSE ranking to account for the uneven performance of the NN on different structures. In addition, in order to avoid overestimating the NN performance, the parameter set in the prediction accuracy calculation algorithm was adjusted and cross-checked by comparing the automatically and manually calculated prediction accuracy. The prediction accuracy of the machine calculation with the final chosen parameter set is slightly lower than that of the manual calculation, ensuring the reliability of the prediction accuracy of the machine calculation.

### Structure prediction of interfacial water based on preliminary training

After preliminary training for 60 epochs using 70 000 data of interfacial water on Au(111) substrate, the loss converges to 0.0025. Structural predictions of high-density and low-density interfacial water by the preliminary trained NN are shown in Fig. 2a and b, indicating good predictions in both the atomic identity and position. Furthermore, the orientation of interfacial water molecules can also be well predicted by the different hydrogen colors in the defined structure representation. Hydrogens colored in light blue, green and brown represent H-flat pose (O–H parallel to the surface), H-up pose (O–H pointing obliquely upward toward the surface) and H-down pose (O–H pointing obliquely downward toward the surface), respectively. Based on 1000 validation data, the prediction accuracy of oxygen and hydrogen reaches as high as 96.2% and 89.9%, respectively. Apparently, this high-resolution interpretation capability of the NN outperforms human experts, who can barely predict all hydrogen positions based on AFM images alone.

To gain insight into the NN performance and further analyse its insufficiency, we analysed the best and worst 10% predictions in order of the network loss. It was found that the loss in nearly all predictions (>99%) is caused by the light-colored dots or shadows on the graph (see Supplementary Fig. S6) that correspond to low-confidence predictions, except for completely failed predictions (<1%). While these light-colored dots and shadows may hinder the accuracy improvements, the predicted structure is still a plausible atomic arrangement and thus can serve as a valuable reference.

To test the generalization ability of the NN preliminarily trained with interfacial water structures on Au(111), the structure prediction was carried out for interfacial water on Pt(111), which has a smaller lattice constant and is more hydrophilic than Au(111) [45]. The test structures include 1000 interfacial water structures at different temperatures and distances from the Pt(111) surface, and their processing method is the same as that of the structures on the Au(111) surface. The structure prediction tested on Pt(111) performs pretty well on both dense and dilute interfacial water (Fig. 2c and d), with a slightly larger loss (0.0030) than that of Au(111) (0.0025). After testing, the prediction accuracies of oxygen atoms and hydrogen atoms are 94.8% and 86.9%, respectively, indicating the generalization ability of the NN and further laying the foundation for transfer learning.

In addition, further analyses of the structure predictions of Pt(111) found that the predicted hydrogen atom positions sometimes shift along the H-bonds compared to the reference (see Supplementary Fig. S7, marked with arrows). Specifically, the hydrogen that should belong to water molecule A was predicted to belong to water molecule B, which is connected to A by hydrogen bonding. We speculate that, due to the weak hydrogen signals in the AFM images, the NN cannot locate hydrogen atoms based solely on image features like other atoms with strong signals, such as oxygen. Instead of detecting atom positions directly from AFM images, the NN uses structural inference to predict the positions of hydrogen atoms based on rules learned from the phase space of the training data. However, water molecules on hydrophobic Au(111) are more likely to be H-up than that on hydrophilic Pt(111), and thus the lack of the relevant structures in the preliminary training data set caused the lower prediction accuracy of hydrogen on Pt(111). Indeed, the prediction mismatch of hydrogen (see Supplementary Fig. S7) was almost completely reduced by retraining NN with the 5000 data of interfacial water structures on Pt(111). Thus, it can be found that data with physics-induced biases are essential and effective for NN to accurately predict one specific system.

### Structure prediction of Na^+^ hydrates with transfer learning

**Figure 3.**
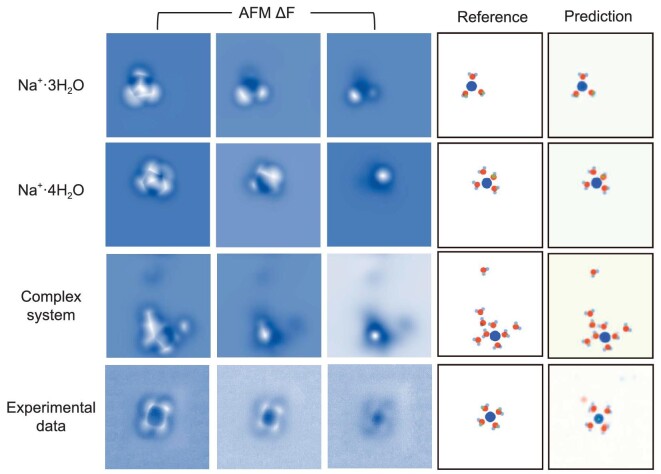
Examples of network prediction from simulated and experimental data for Na^+^ hydrates. Rows 1 and 2 are the two basic structures, Na^+^⋅3H_2_O and Na^+^⋅4H_2_O, selected from the validation data set, respectively. Row 3 is the more complex sodium hydrate structure in the testing data to examine network transferability. Row 4 is the experimental data. Columns 1–3 are AFM images (input data) with increasing tip-sample distance. Column 4 is the reference a-vdW sphere representation of the structure (label) and Column 5 is the prediction from the network. For the row of Experimental Data, the AFM images are obtained at different tip heights of 25 pm (left panel), 70 pm (middle panel) and 120 pm (right panel). The tip height of the experimental AFM images is referenced to the STM set point on the NaCl surface (100 mV, 50 pA). Adapted with permission from Peng *et al.* 2018. *Nature* 557 (7707): 701–5. The reference structure was validated by relaxing the predicted structure and comparing the AFM images simulated based on it with the experiment. The red and indigo spots represent oxygen and sodium atoms, respectively. The light blue, green and brown spots represent hydrogen atoms at the same height as above and below the oxygen atoms, respectively.

For the interfacial Na^+^ hydrates system, we used transfer learning to efficiently train NN by loading the parameters of the preliminarily trained NN. Na^+^⋅nH_2_O (*n* = 3,4) on NaCl(001) was chosen as the basic structure to explore the phase space of the Na^+^ hydrates system, whose structures were randomly located within the detection window. Unlike interfacial water, AFM simulations of the ionic hydrate structures were performed using electrostatic potentials obtained from DFT calculations. After training for 50 epochs with data based only on 1500 Na^+^ hydrate structures, the loss converged to 0.0009 and the NN prediction performed very well (Fig. 3, the first and second rows; Supplementary Fig. S5). The predicted accuracies for sodium, oxygen and hydrogen calculated on 500 validation data are 96%, 95.4% and 85%, respectively.

To test the transferability of the retrained NN via transfer learning, this NN was applied to predict the structure of more complex Na^+^ hydrates with more than four water molecules. The predicted structure matches the actual structure remarkably well (Fig. 3, the third row; Supplementary Fig. S5) and the prediction accuracies for sodium, oxygen and hydrogen atoms based on the testing data set of 1000 complex Na^+^ hydrates are 82.3%, 91.7% and 72.9%, respectively. In addition, experimental AFM images of Na^+^⋅4H_2_O were used for structure prediction to test the effectiveness of the NN. The corresponding predicted structures agree very well with the DFT-optimized geometry (Fig. 3, the fourth row) and its simulated AFM images are in perfect agreement with the experimental result (see Supplementary Fig. S8). We note that the light red dots in the upper left corner of the Na^+^⋅4H_2_O in the prediction can be discarded, as it is a low-confidence prediction. Therefore, it can be determined that NNs can provide valuable structure predictions even for complex Na^+^ hydrate AFM images and experimental AFM images. Moreover, following the training procedures, our NN gives a good prediction for experimental data of K^+^ hydrates, which indicates the general applicability of our method (see Supplementary Figs S10 and S11).

Generally speaking, the water molecules in Na^+^ hydrate can be divided into those around Na^+^ and those above Na^+^ according to their positions relative to Na^+^. To further understand the prediction performance of NNs on different water molecules, the prediction accuracy of these two types of water was also analysed (Supplementary Fig. S9) and significant differences were found between them. For water molecules around Na^+^, the prediction of oxygen and hydrogen is good. Conversely, atoms in water molecules above Na^+^ are difficult to be predicted by NNs. This poor prediction may be due to the fact that the actual height of such systems exceeds one layer and thus goes beyond the trained 2D representation, making NN parsing difficult.

### Validity assessment of the transfer learning

**Figure 4.**
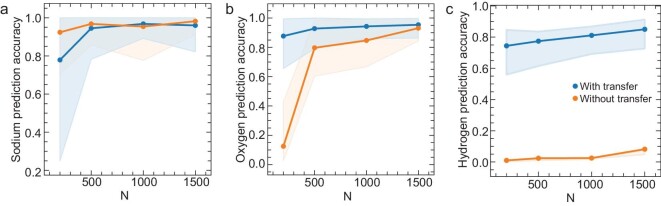
The prediction accuracy of a neural network as a function of the size of the training data. (a), (b) and (c) are the positional accuracies of sodium, oxygen and hydrogen atoms, respectively. The blue and orange lines represent the training process with and without pretrained parameter loading, respectively. The solid line is calculated by counting the accuracy in all validation data, while the upper and lower bounds of the color patches are calculated from the data at the top and bottom 20% of the loss ranking, respectively.

To evaluate the effectiveness of transfer learning, we used different training data sets (with different data volumes, $N\ = \ $200, 500, 1000 and 1500) to train NNs with or without transfer learning. The prediction accuracy for these different training segments was calculated by using the same validation data set containing 500 Na^+^ hydrate structures, and the best 100 and worst 100 data sorted by loss were calculated as the upper and lower bounds of the error bar (Fig. 4). When *N*$\ge $ 500, the NN trained with transfer learning has a higher prediction accuracy of all atomic species than that without transfer learning (Fig. 4a and b, blue line). The prediction accuracy of the hydrogen based on the directly trained NN without transfer learning is almost zero (Fig. 4c, orange line) and only small patches of light gray near oxygen are presented (Supplementary Fig. S12). However, with transfer learning of the preliminarily trained NN, the high prediction accuracy of hydrogen can be achieved with only a few thousand data.

Notably, when *N*$\ < $ 500, the directly trained NN shows better performance in Na^+^ prediction than that with transfer learning. We speculate that this phenomenon is due to two aspects. One is that the signal for Na^+^ in the AFM image is the strongest, which is easy for a blank NN to learn. The other is that the retraining of the preliminarily trained NN via transfer learning requires sufficient Na^+^ hydrate data to get rid of previous local minima. Furthermore, this drawback of transfer learning soon disappears as *N* increases to 500, while the prediction accuracy of transfer learning does not increase much when *N* > 1000, suggesting that only a few hundred data are required to train an accurate NN in this case. Therefore, it can be concluded that the NN preliminarily trained with the interfacial water data can be transferred to the ionic hydrate system through transfer learning, so that it can be realized to train the NN with only a small amount of data to obtain the ability of high-precision prediction for more complex systems.

## DISCUSSION

In this work, we presented an economic method to identify the atomic structure of interfacial ionic hydrates from AFM images using transfer learning that solves the huge demand for high-cost DFT calculation in the data preparation. Through empirical potential-based MD simulations and AFM simulations based on simple point charges and L–J interactions, we first sacrificed a little precision to generate a large number of AFM images of interfacial water and trained the NN of a 3D U-Net with these data to capture the atomic structure of the H-bond network. Next, by retraining with only a few thousand hydrate structure data through transfer learning, the NN can achieve accurate structure prediction of all atoms in hydrates, outperforming the ability of human experts. More importantly, after comparing the performance of NN training on the hydrate structure with or without transfer learning, we verified the feasibility and validity of the transfer-learning method in reducing the demand for computational resources. In addition, this method is transferable to different systems, as demonstrated by the good predictions for both interfacial water on different substrates (Au and Pt surface) and complex Na^+^ hydrates with different water-molecule numbers.

Importantly, this work is a good start in high-resolution atomic structure prediction based on AFM images. There is room for improvement; for instance, experimental errors such as noise distribution and tip drifting need to be handled more carefully to further improve the prediction accuracy. With the rapid application of physical laws and geometry symmetries to informed NNs [24,46] in general drug *de novo* design [47], biomolecular structure prediction [48,49] and molecule surface potential prediction [50,51], we believe that our approach can determine 3D structures (e.g. ice) by introducing those NN architectures. Finally, it is worth noting that our proposed ML method with transfer learning can also be extended from efficient structure prediction for AFM imaging to a broad range of applications for interpreting other experimental measurements [52], such as scanning tunneling microscopy (STM) [53], scanning electron microscopy (SEM) [30] and transmission electron microscopy (TEM) [54].

## METHODS

### NN structure

The NN for structure prediction from AFM images was a standard 3D U-Net [42] model, a CNN widely used for biological and clinical image segmentation [43]. It consists of an encoder part for feature extracting, a decoder part for converting input (AFM images) to output (visual representation) and skip connections between the encoder and decoder parts, effectively preventing the feature loss of H-atoms during the encoder process (see details in SI Section 1).

The loss function for this network is the MSE:


\begin{eqnarray*} {\rm{MSE }}\left( {y,\tilde{y}} \right) = \frac{1}{N}\ \mathop \sum \limits_{i = 1}^n {\left( {{y}_i - {{\tilde{y}}}_{\rm{i}}} \right)}^2, \end{eqnarray*}

where *N* is the number of images, *n* is the number of pixels in each image, and *y* and $\tilde{y}{\rm{\ }}$are the NN output and data labels, respectively. For the gradient descent optimization, the Adaptive Moment Estimation (Adam) optimizer was used [55].

### MD simulations

A vapor–liquid–solid system was constructed and the periodic boundary conditions were applied in all directions. Water molecules are represented by a rigid SPC/E model [56]. Au/Pt atoms, fixed throughout the simulation, interact with water molecules only through L–J interactions [57]. The Lorentz−Berteloth Combination Rules [58] are used (more details are in SI Section 2).

### Simulations of AFM images

The AFM images were simulated using a molecular mechanics model based on methods described in Refs [21] and [22]. We perform AFM simulations to model the CO tip based on the probe-particle-tip model with the following parameters: effective lateral stiffness *k* = 0.50 N/m, atomic radius *R_c_* = 1.661 Å and *Q* = −0.05 e (e is the elementary charge). These parameters can effectively reproduce most of the important features of experimental AFM images. It is important to note that small changes in the simulation parameters for training data do not significantly change the predictions on the experimental data. The tip height is defined as the distance between the outmost metal atom of the tip and the average height of O atoms of water molecules. For the interfacial water on the metal substrate, the charge distribution is described as a point charge on each atom, while for Na^+^ and K^+^ hydrates, the electrostatic potentials used in the AFM simulations are obtained from DFT calculations, where the substrate has negligible influence on the AFM image and is ignored to reduce computational costs (see SI Section 3 for details). The parameters of the L–J pairwise potentials for all elements are listed in Supplementary Table S3.

### AFM experiments

The experimental images of Na^+^ hydrates were taken from Ref. [14]. The experimental method for K^+^ hydrated on the Au surface is described in detail here. The Au(111) single crystal was purchased from MaTeck. The Au(111) surface was cleaned by repeated Ar^+^ ion sputtering at 1 keV and annealing at ∼700 K for multiple cycles. The SAES alkali-metal dispensers were degassed before evaporation (current *I_K_* = 6.5 A, *t* = 2 min). The alkali-metal atoms were deposited on the clean Au(111) surface at room temperature (deposition current *I_K_* = 6.3 A, *t* = 1 min). The ultrapure H_2_O (Sigma Aldrich, deuterium-depleted, 1 ppm) was used and further purified under vacuum by three to five freeze-and-pump cycles to remove remaining gas impurities. The water molecules were deposited on the Au(111) surface at 120 K. All the experiments were performed by using a non-contact AFM system (Createc, Germany) at 5 K using a home-made qPlus sensor equipped with a tungsten (W) tip (spring constant k_0_ ≈ 1800 N·m^−1^, resonance frequency *f*_0_ ≈ 28.7 kHz and quality factor *Q* ≈ 100 000). All the AFM frequency shift (Δ*f*) images were acquired by using the CO-terminated tips in frequency modulation and constant-height mode. The oscillation amplitude of experimental AFM imaging is 100 pm.

## DATA AVAILABILITY

The simulated AFM data of interfacial water and ionic hydrates are available in Zenodo at https://doi.org/10.5281/zenodo.7483100.

## Supplementary Material

nwac282_Supplemental_File
