# Supplementary material for: Machine learning-aided atomic structure identification of interfacial ionic hydrates from AFM images
Source: Natl Sci Rev. 2022 Dec 14;10(7):nwac282. doi: 10.1093/nsr/nwac282 (PMC10232042; doi:10.1093/nsr/nwac282)
Supplement: nwac282_Supplemental_File [file nwac282_supplemental_file.pdf]

<u>Supplementary Information</u>: Machine Learning Aided Atomic Structure Identification of Interfacial Ionic Hydrates from AFM Images

Binze Tang[1,2†], Yizhi Song[1,2†], Mian Qin[2†], Ye Tian[1,2†], Zhen Wei Wu[3], Ying Jiang[1,2,4,5,6*], Duanyun Cao[7,8*], Limei Xu[1,2,4,6*]

[1]International Center for Quantum Materials, Peking University, Beijing, 100871, China

[2]School of Physics, Peking University, Beijing, 100871, China

[3] Institute of Nonequilibrium Systems, School of Systems Science, Beijing Normal University, 100875 Beijing, China

[4] Collaborative Innovation Center of Quantum Matter, Beijing, 100871, China

[5] CAS Center for Excellence in Topological Quantum Computation, University of Chinese Academy of Sciences, Beijing, China

[6] Interdisciplinary Institute of Light-Element Quantum Materials and Research Center for Light-Element Advanced Materials, Peking University, Beijing 100871, China

[7]Beijing Key Laboratory of Environmental Science and Engineering, School of Materials Science and Engineering, Beijing Institute of Technology, Beijing, 100081, China

[8]Beijing Institute of Technology Chongqing Innovation Center, Chongqing, 401120, China

[†]These authors have contributed equally to this work and share co-first authorship.

[*]Corresponding author. E-mail: yjiang@pku.edu.cn (Y. J.), dycao@bit.edu.cn (D. C.), limei.xu@pku.edu.cn (L.-X.)

This PDF file includes:

Section 1. Neural Network Structure.

Section 2. Calculation Details.

Section 3. Simulated AFM image of interfacial water with/without Au substrate

Section 4. Structure Sampling.

Section 5. Training Data Augmentation.

Section 6. Structure Representation.

Section 7. Training Process.

Section 8. NN Prediction of Interfacial Water.

Section 9. NN Prediction of hydration Sodium.

Section 10. Bad prediction of NN on interfacial water (Au surface) data.

Section 11. Correction of the hydrogen atom shift by transfer learning.

Section 12. Prediction for experimental AFM images of $Na^{+}\cdot 4H_2O$.

Section 13. Prediction for Na hydrates whose water molecules are at different positions.

Section 14. Structure prediction of K^{+} hydrates with Transfer Learning

Section 15. The prediction performance of NN with or without transfer learning.

Section 16. Object detection for each atom.

Figure S1. 3d U-Net architecture.

Figure S2. The simulated AFM image of interfacial water with/without Au substrate.

Figure S3. Structure representation.

Figure S4. Detailed prediction and reference representations of Fig. 2.

Figure S5. Detailed prediction and reference representations of Fig. 3.

Figure S6. Bad prediction of NN on interfacial water (Au surface) data.

Figure S7. Hydrogen atom shift in NN prediction of the water structure on Pt surface.

Figure S8. Validation of the predicted $Na^{+}\cdot 4H_2O$ structure according to the experimental AFM images.

Figure S9. Prediction for Na hydrates whose water molecules are at different

positions.
Figure S10. Examples of network prediction of K^+ hydrates simulated and experimental data.
Figure S11. Validation of the predicted K^+ hydrates structure.
Figure S12. Prediction of Na hydrates by NN with or without transfer learning.
Figure S13. The prediction accuracy error bar in Fig. 4 with the manually collecting data.
Table S1. Force field parameters of SPC/E water, Au and Pt surface for interfacial water simulation.
Table S2.1. Force Field Parameters for Sodium Chloride.
Table S2.2. Force Field Parameters for Potassium Chloride
Table S2.3. Force Field Parameters for polarizable water model based on SPC/ε model.
Table S3. Parameters of L-J pairwise potentials for AFM simulations.
Table S4. Parameters used in atom detection.

1. Neural Network Structure

The Neural Network (NN) for structure prediction from atomic force microscopy (AFM) images was a standard 3D U-Net[1] model, a CNN widely used for biological and clinical image segmentation[2]. It consists of an encoder part for feature extracting, a decoder part for converting input (AFM images) to output (visual representation), and skip connections between the encoder and decoder parts, effectively preventing the feature loss of H-atom during the encoder process (fig. S1). 10 of the gray scale AFM images at different tip-sample distances are stacked to a 3D image as the input to the network. The size of the input data is $1 \times 10 \times 128 \times 128$ where each number represents the channel, depth, height and width respectively. The output of the network is a 3 channels 2D image of size $3 \times 128 \times 128$ where each number represents the channel, height and width respectively. Each block in the encoder part contains two $3 \times 3 \times 3$ convolutional layers, each layer is preceded by a Group Normalization (GN) layer and followed by a leaky rectified linear unit (L-ReLU). And a maxpooling is added at the end of each block. The first two maxpoolings are $1 \times 2 \times 2$ in size to keep the AFM stack depth at 10, and the next two maxpoolings are $2 \times 2 \times 2$ in size to reduce the data depth to 2. In the decoder part, each block consists of an interpolation upsampling of the size of maxpooling in the corresponding block in the encoder, followed by two $3 \times 3 \times 3$ convolution layers, each of which is preceded by a GN layer and followed by a L-ReLU. Skip connections between layers with the same size in the encoder and decoder part provide the decoder with essential high-resolution features. After the decoder, there are two $3 \times 3 \times 3$ convolution layers and two 3×3 convolution layers. The data was flattened before the 3×3 convolution. Except for the last layer using ReLU, other convolution layers use L-ReLU. The slope of the L-ReLU is 0.001.

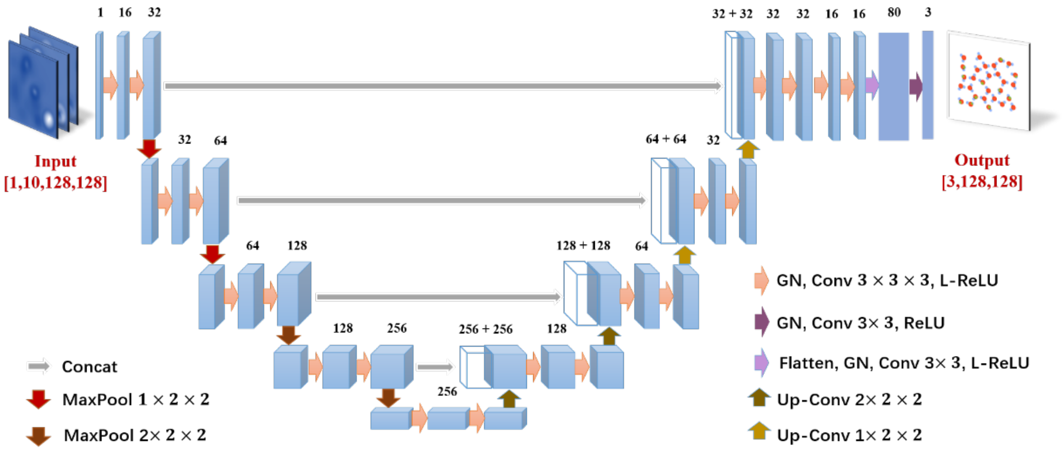

Figure S1 | 3d U-Net architecture. Schematic illustration of the model architecture. Blue cuboids and rectangles represent 3D and 2D feature maps, respectively. The number of channels is denoted above each feature map.

2. Calculation Details

2.1 The MD simulations of interfacial water.

The MD simulations of interfacial water are performed in the NVT ensemble using the LAMMPS software packages[3]. In the constructed system of the interfacial water on Au(111) surface, the size of the entire box is about $5.0 \times 5.0 \times 7.5\ nm^3$, the Au(111) surface modeled by a six-layer slab is fixed at the bottom of the box, the water tank with a size of about $5.0 \times 5.0 \times 3.0\ nm^3$ is placed right above the surface and the rest of the box is vacuum space. For the system of interfacial water on the Pt surface, we only replace the surface to Pt (111) and adjusted the box size according to the lattice difference between Pt and Au surface. The detailed force field parameters are listed in Table S1. MD simulations over a wide temperature range from 140K to 300K ($40K$ a step) , were performed to sample various water phase spaces sufficiently. The simulation timestep is 1 fs and the total simulation time at a given (N, V, T) is $5\ ns$ where the first $2\ ns$ is run for structure relaxation.

2.2 The MD simulations of ion hydrates.

The MD simulations of Na^+ /K^+ hydrates were performed using the AMBER16 software package[4] with polarizable force fields[5][6] for Na^+ hydrates and the recently optimized force fields for K^+ hydrates[7]. The effectiveness of the force field for Na^+ hydrates has been demonstrated in our previous publication[8]. The force field parameters are shown in Table S2.1-2.3. A four-layered NaCl/KCl crystal ($18 \times 18 \times 4$ atomic number) with a (001) surface was used to support Na^+/K^+ hydrates. The MD simulation for each system was run no shorter than $1\ ns$ for adequate configurations. The time step was set to 1 fs and the temperature is controlled using Langevin dynamics with a collision frequency of $0.1 ps^{-1}$. The bottom layer of the NaCl/KCl crystal was constrained by a force constant of $2000\ kcal/(mol \cdot Å^2)$, and the periodic boundary conditions were applied in all directions. The SHAKE algorithm was used to constrain all bonds involving hydrogen atoms[9] and a cutoff of 1.0 nm was used for van der Waals interactions. A long-range dispersion correction based on an analytical integral,

assuming an isotropic, uniform bulk particle distribution beyond the cut-off, was added to the van der Waals energy and pressure[4].

2.3 DFT calculation.

We calculate the electrostatic potential of Na^{+}/K^{+} hydrate at Au (111) surface using DFT within the generalized gradient approximation of Perdew-Burke-Ernzerhof[10] (PBE-GGA), which has been shown to give good results for H_2O hydrogen bonding[11] DFT calculations were performed using the Vienna ab initio simulation package (VASP)[12]. Projector augmented wave pseudopotentials were used with a cut-off energy of 550 eV for the expansion of the electronic wave functions. Van der Waals corrections for dispersion forces were considered by using the optB86b-vdW functional[13] [14].

Table S1 Force field parameters of SPC/E water, Au and Pt surface for interfacial water simulation

Element	q [e]	σ [Å]	ε [Kcal/mole]
H	+0.4238	0.0	0.0
O	-0.8476	3.166	0.1553
Au	0.0	2.629	5.29
Pt	0.0	2.535	7.8

Table S2.1 Force Field Parameters for Sodium Chloride

Model	q [e]	λ_c	σ [Å]	(ε/k_B) [K]
Na	+1	0.885	2.52	17.44
Cl	-1	0.885	3.85	192.45

Table S2.2 Force Field Parameters for Potassium Chloride

Model	q [e]	σ [Å]	ε[KJ/mol]
K	+1	2.791	2.223

Cl	-1	5.029	0.02742

Table S2.3 Force Field Parameters for polarizable water model based on SPC/ε model

model	r_{OH} [Å]	Θ [deg]	q_H (q_O) [e]	σ [Å]	(ε/k_B) [K]
SPC	1	109.47	0.445 (-0.890)	3.1785	84.9

Table S3 Parameters of L-J pairwise potentials for AFM simulations

Element	ε[meV]	r [Å]
H	0.680	1.487
O	9.106	1.661
Na	10.0	1.40
K	10.0	1.50

3 Simulated AFM image of interfacial water with/without Au substrate

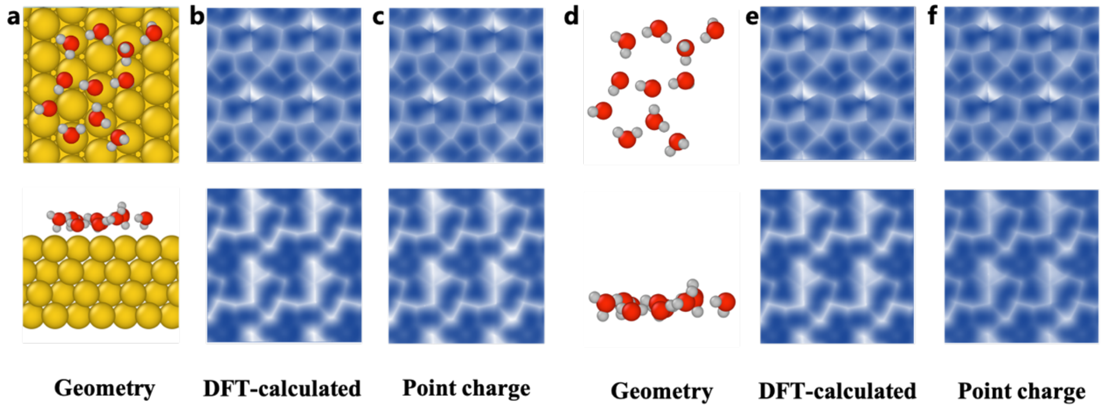

Figure S2 | The simulated AFM image of interfacial water with/without Au substrate. (a and d) The atomic structure of the interfacial water on Au substrate and the same structure without Au substrate, respectively (upper panel: top view; lower panel: side view). The red, white and golden spheres represent O, H and Au atoms, respectively. (b and c) The simulated AFM images of structure (a), where the electrostatic potential surface is obtained based on DFT (b) and point charge model (c), respectively. (e and f) The simulated AFM images of structure

(d), where the electrostatic potential surface is obtained based on DFT (e) and point charge model (f), respectively. It can be found that the effect of substrates on the AFM images was negligible, and the simulated AFM images with and without substrates are nearly identical.

4 Structure Sampling

We note that the detection window (Fig. 1a, the first panel) in size of $2.5 \times 2.5 \times 0.3 nm^3$ is large enough to cover the various interfacial water structures in the XY plane, and ensure that the AFM can only probe one layer along the Z direction. Thus, all of the structures are restricted in the window and their corresponding simulated AFM images have the same detection range in size $2.5 \times 2.5\ nm^2$.

For acquiring sufficient interfacial water structure, the detection window is initially settled at the first water layer and then slides in the direction parallel and perpendicular to the substrate. In XY plane, the number of steps of the detection window is 2 and the stride is $0.5\ nm$ for each step. While in Z direction, the number of steps and stride is 1 and $0.5\ nm$, respectively. To ensure the variety of the data, the water molecules within the distance δb_x and δb_y from the window boundary are neglected during each detection. The distance δb_x and δb_y are settled randomly, but can guarantee that at least one water molecule remains in the detection window. The configurations used for data acquisition is sampled from the MD simulations trajectories with a time interval of $10\ ps$.

5 Training Data Augmentation

To make the network more robust to experimental data and avoid overfitting, the input data is processed as follows.

a) Nosie. For each data, a random uniform noise or a gaussian noise is randomly selected and added to all the images.

   A random uniform noise with an amplitude in a range of $[-\delta P, \delta P]$ is added to each image,

$$\delta P = c(P_{max} - P_{min}), \qquad \text{(S1)}$$

   where P_{max} and P_{min} is the maximum and minimum pixel value of an image,

and c is the amplitude ratio.

A gaussian noise with an amplitude in a distribution P_G is added to each image,

$$P_G(x) = 255 \times c' \frac{1}{\sqrt{2\pi}\sigma} \exp\left(-\frac{x^2}{2\sigma^2}\right), \qquad \text{(S2)}$$

Where c' is the amplitude ratio, $\sigma = 1$.

For training the interfacial water data, c=0.03, c'=0.01. When transfer learn the Na^+/K^+ hydrates for experimental prediction, c and c' is randomly selected from a uniform distribution in range [0, 0.2] and [0, 0.15], respectively.

b) Pixel Shift. In experimental AFM images, for some technical reasons, the positions of specific structural features relative to the scanning boundary at different tip-sample distances could not remain exactly the same. Each image in our data is shifted a few pixels away in $\pm x$ and $\pm y$ directions. The movement distance is a random value of less than three pixels.

c) Cutout. Experimental AFM images inevitably have tip shifts, where there is something fuzzy. To simulate this, a rectangle with a gray value of the average of the entire image and a size less than 500 pixels is generated on each image to cover the image underneath.

d) Distance Randomization. 20 AFM images at 0.1 Å intervals are prepared. First, 15 consecutive images are selected. Then, 10 images are randomly selected from these to form a stack.

6 Structure Representation

We design a new structure representation named advanced vdW (a-vdW) spheres as the neural network output/ data label. As shown in fig. S3, it composed of three grayscale images representing atom type, positive charge and negative charge. They are the same size and are all the projection of the structure onto the $x - y$ plane. Since the interfacial structure is restricted in a box with a height of only 0.3 nm, its information

in the z direction is negligible, and the projection of all the atoms is sufficient to represent the structure to some extent. In our representation, each atom is described as a sphere and projected onto the $x - y$ plane in height order. If two atoms overlap, the higher atom cover the lower one. In atom-type images, the diameter d and grayscale g_1 of atoms are related to their LJ potential parameters σ and ϵ. Here, in order to differentiate different elements for easier identification, ϵ of each element is scaled and transformed,

$$d = \sigma - k_1\left(1 - \left(\frac{\epsilon - \epsilon_{min}}{\epsilon_{max}}\right)^{\frac{1}{2}}\right), \qquad \text{(S3)}$$

$$g_1 = 255 \times k_2\left(1 - \left(\frac{\epsilon - \epsilon_{min}}{\epsilon_{max}}\right)^{\frac{1}{2}}\right), \qquad \text{(S4)}$$

where $\epsilon_{min}\ and\ \epsilon_{max}$ are the minimum and maximum ϵ values among all elements, respectively, and $k_1 = 0.6$ and $k_2 = 0.7$ are the scale factors. All the LJ potential parameters are chosen from the OPLS Force Field[15]. For positive and negative charge images, the diameter of atom is defined as above, and the grayscale g_2 is scaled by charge,

$$g_2 = 255 \times \left(1 - \frac{|q|}{|q_{max}|}\right), \qquad \text{(S5)}$$

where q is the charge of the atom and q_{max} is the charge of the most charged atom in the structure.

If we consider these three images as three channels of a color image and combine them, we get a colormap where each atom has a different color. According to the definition of this structure representation described above, the light blue, green, and brown hydrogens represent H-flat Pose (O-H parallel to the surface), H-up Pose (O-H pointing obliquely upward to the surface), and H-down Pose (O-H pointing obliquely downward to the surface), respectively. This representation synthesizes the basic characteristics and position information of atoms. Similar to the structure atom map shown by a popular visualization software Ovito[16] (see fig. S3), this representation is highly readable and understandable.

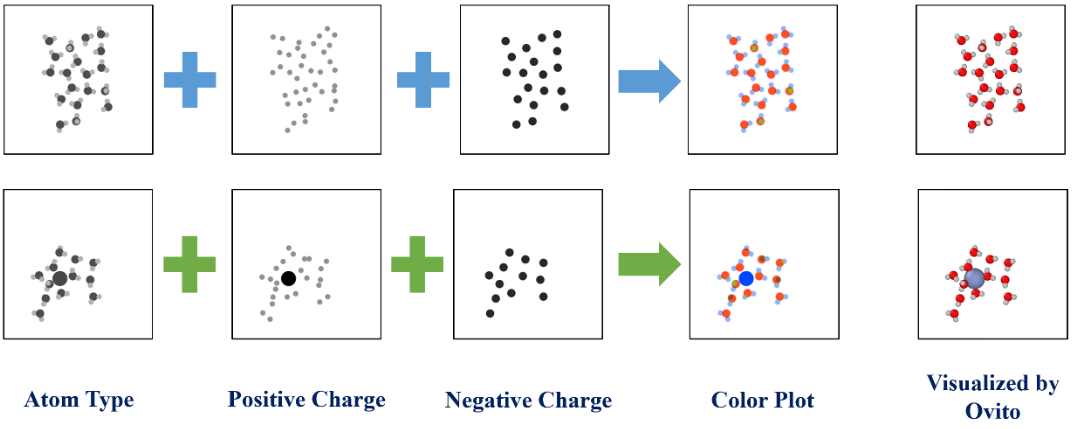

Figure S3 | Structure representation. The first three columns are grayscale representations of atom type, positive charge and negative charge, respectively. A colormap is the combination of three grayscale representations in RGB channels. The last column is a structure atom map visualized by the popular software Ovito for comparison. The first and second rows represent interfacial water and ion hydrate, respectively.

7 Training Process

The loss function is mean square error (MSE) and the optimizer for gradient descent is the Adaptive Moment Estimation (Adam)[17]. For the training of interfacial water data, the learning rate is 0.001 in the first 30 epochs, and then decreases to 0.0001 for the next 30 epochs. For the transfer learning of Na^+ hydrates, trained network parameters from the interfacial water dataset are used. The learning rate for the last two 2D 3×3 convolution layers and other layers are 0.001 and 0.0001, respectively. The loss converges after 60 epochs of training. The batch size we use throughout the training is 8.

8 NN Prediction of Interfacial Water

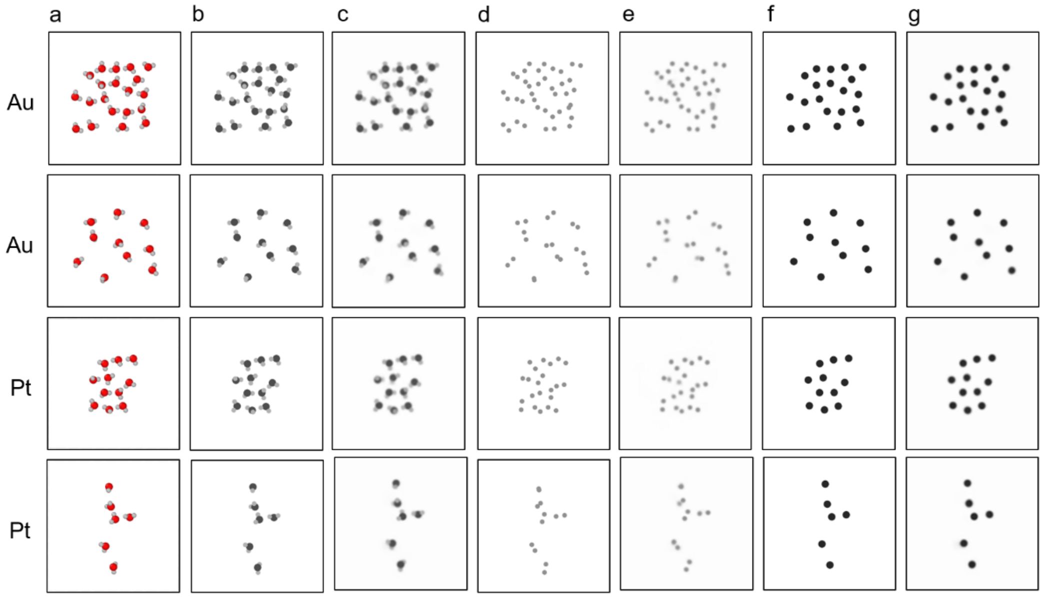

Figure S4 | Detailed prediction and reference representations of Fig. 2. a, The atomic model presented by OVITO. **b, d, f,** The reference representations of structure **a**, which is atom type, positive charge and negative charge, respectively**. c, e, g,** The representations predicted by the NN for structure **a**, which is also atom type, positive charge and negative charge, respectively**.**

9 NN Prediction of hydration Sodium

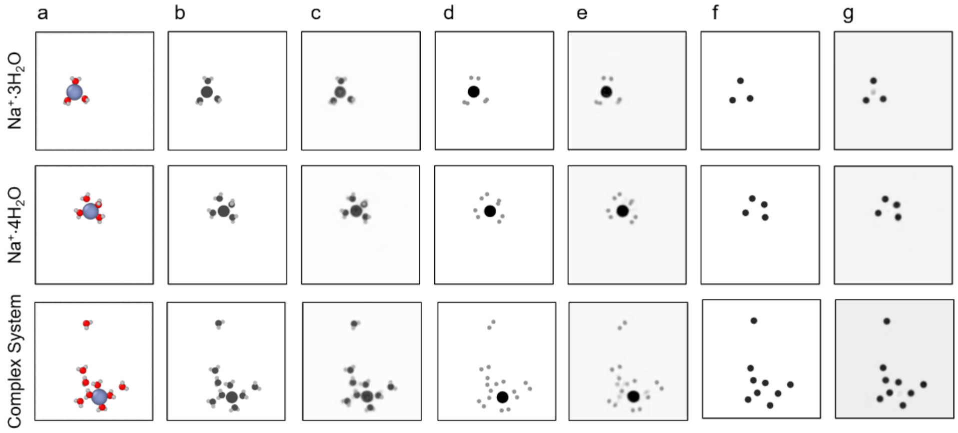

Figure S5 | Detailed prediction and reference representations of Fig. 3. a, The atomic model presented by OVITO. **b, d, f,** The reference representations of structure **a**, which is atom type, positive charge and negative charge, respectively**. c, e, g,** The representations predicted by the NN for structure **a**, which is also atom type, positive charge and negative charge, respectively**.**

10 Bad prediction of NN on interfacial water (Au surface) data

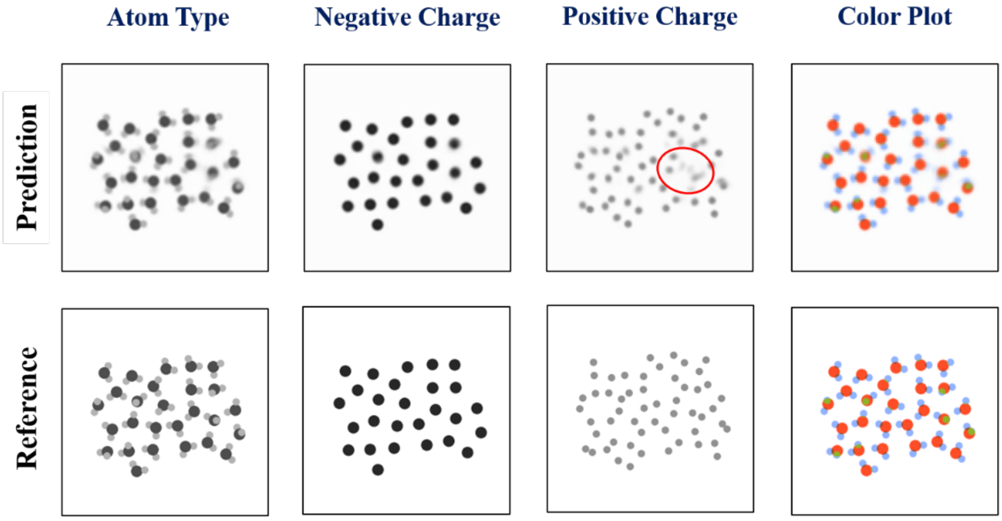

Figure S6 | Bad prediction of NN on interfacial water (Au surface) data. The representations of structure **a**, which is atom type, negative charge, positive charge, and color plot, respectively (the upper panels, predicted by NN; the lower panels, reference representations).

11 Correction of the hydrogen atom shift by transfer learning

After training the NN using data of interfacial water on Au (111) substrate, we test the transferability of NN using the data of interfacial water on Pt (111) substrate (testing data). Manually examination of the prediction of NN on testing data revealed that some of the predicted hydrogen atom positions drifted along hydrogen bonds compared to the reference. As can be seen from fig. S7 (the middle plot), the hydrogen shifts are marked with arrows, and the misdescription of hydrogen atom is marked with the circle. After re-training (transfer learning) the NN using 5000 data of interfacial water on Pt (111) substrate, the hydrogen shifts almost disappeared (fig. S7, the right plot).

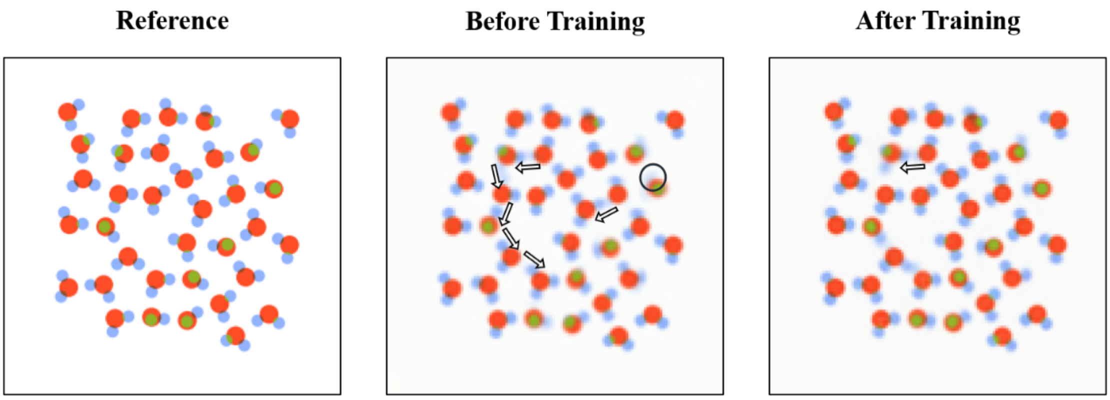

Figure S7 | Hydrogen atom shift in NN prediction of the water structure on Pt surface. The first plot is the reference structure; the second and third plots are the colormaps predicted by the NN before and after training on the interfacial water structures on Pt surface. The arrows indicate the directions of the displacement of hydrogen atoms, and the circles indicate the mispredictions of the hydrogen atom compared to the reference.

12 Prediction for experimental AFM images of $Na^{+}\cdot 4H_2O$

Before prediction, the experimental AFM images to be predicted should be pre-processed. They should be padded or cut to the same size as the detection space of the training data, so as to ensure that the size of the imaging of a specific molecule relative to the detection space remains unchanged. Here, the detection space of experimental AFM images to be predicted is $1.8\,nm \times 1.8\,nm$ and the image size is 240×240 pixels, which should be padded to 333×333 pixels, since the detection space of the training data is $2.5\,nm \times 2.5\,nm$. Then, the images are resized to 128×128 pixels as the input data. In addition, to avoid the influence of experimental noise on the NN prediction, we adjust the noise amplitude of the data augmentation $c = 0.01$ during the NN training process for ionic hydrates data.

According to the prediction of NN of experimental data (Fig. 3, the prediction in last row), we create a $Na^{+}\cdot 4H_2O$ structure on NaCl substrate and simulate the AFM images. Comparing the simulated (fig. S8, second row) and experimental (fig. S8, first row) AFM images, we can conclude that the NN can give a good prediction for Na hydrates experimental data. To be noticed, the unsymmetric feature of the experimental AFM image was caused by the tip drift during the image scanning. Specifically, it took a long

time (about 1 hour) to complete the scanning of one AFM image. In such a long time, the tip would drift to a certain extent, and the AFM image would appear unsymmetric.

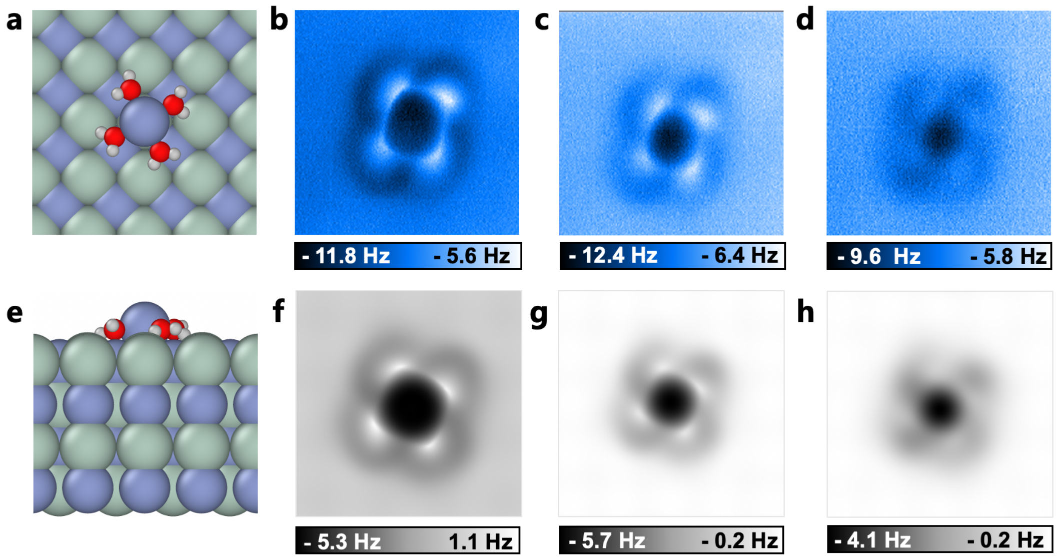

Figure S8 | Validation of the predicted $Na^+ \cdot 4H_2O$ structure according the experimental AFM images. a and **e,** Atomic model (a: top view; e: side view) according to the NN prediction in Fig. 3. The red, white, lilac and cyan spheres represent O, H, Na and Cl, respectively. **b - d,** Experimental AFM images of the $Na^+ \cdot 4H_2O$ at different tip heights of 25 pm (b), 70 pm (c) and 120 pm (d). **f - h,** Simulated AFM images of the $Na^+ \cdot 4H_2O$ at increasing tip heights from left to right. The tip heights in b-d are referenced to the STM set point on the NaCl surface (100 mV, 50 pA). The tip heights in f-h are defined as the vertical distance between the apex atom of the metal tip and the outmost atom of NaCl substrate. The oscillation amplitude of experimental and simulated images is 100 pm.

13 Prediction for Na hydrates whose water molecules are at different positions

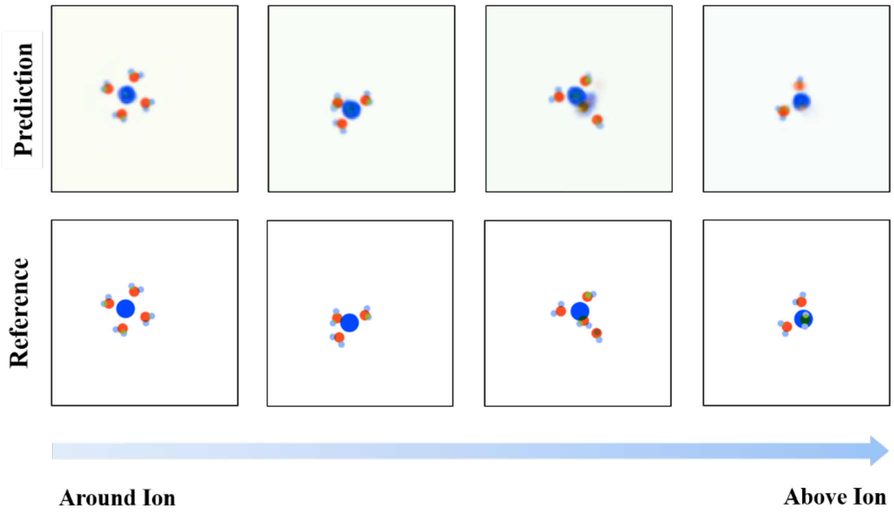

Figure S9 | Prediction for Na hydrates whose water molecules are at different positions. The representations of Na hydrates, from left to right where one of the water molecules posited from around the ion to above the ion (the upper panels, predicted by NN; the lower panels, reference representations).

14 Structure prediction of K^+ hydrates with Transfer Learning

To verify the general applicability of our transfer learning method, we carried out further investigations on K^+ hydrates, following the same operation as for Na^+ hydrates. In particular, thousands of $K^+ \cdot nH_2O$ ($n>3$) data were prepared by simulation for NN transfer learning. To reproduce most of the important features of experimental AFM images, the AFM simulation parameter of charge at the tip apex was used Q = -0.20 e. After 50 epochs of training, the NN shows quite good performance on the K^+ hydrates testing data (fig. S10). The predicted accuracies for K, O and H (calculated from 700 simulated testing data) are 99.9%, 96.3%, and 81.4%, respectively. This is a good demonstration of the robustness and general applicability of our method.

Prediction of experimental AFM images of K^+ hydrates on Au surface is presented in the fourth row of fig. S10 below. Validation of the predicted K^+ hydrates structure is shown in fig. S11, which is verified by the consistency between the experimental AFM image and the simulated AFM image of the NN-predicted structure after DFT relaxation (fig. S11). It is worth mentioning that since the experimental image used to predict the structure is a part of a large interfacial hydrogen bond network, the simulated AFM

images of the predicted structure near the boundary where the periodic boundary condition was applied, show some differences compared with the experimental image. Nevertheless, the AFM simulation of the predicted structure agrees well with the experimental result, demonstrating the high predictive power of our method for the experimental AFM results.

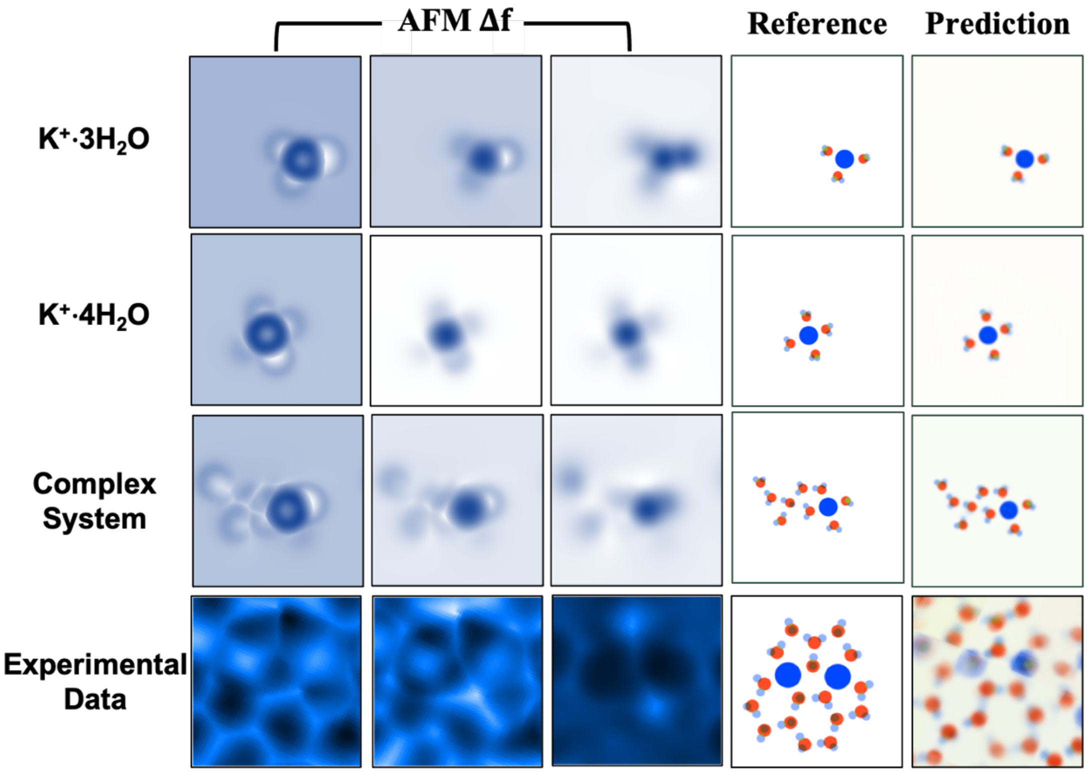

Figure S10 | Examples of network prediction of simulated and experimental data for K^+ hydrates. Rows 1-3 are the $K^+\cdot 3H_2O$, $K^+\cdot 4H_2O$, and the complex K^+ hydrate structure, selected from the calculated test dataset, respectively. Row 4 is the experimental data. Columns 1-3 are AFM images (input data) with increasing tip-sample distance. Column 4 is the reference a-vdW sphere representation of the structure (label), and column 5 is the prediction from the network. For the row of Experimental Data, the AFM images are obtained at different tip heights of -235 pm (left panel), -205 pm (middle panel) and -145 pm (right panel). The tip heights in experimental data are referenced to the STM set point on the K^+ cation (100 mV, 10 pA). The reference structure was verified by relaxing the predicted structure by DFT and comparing the AFM images simulated based on it with the experiment images. The red and indigo spots represent oxygen and kalium atoms, respectively. The light blue, green, and brown spots represent hydrogen atoms at the same height as, above and below the oxygen atoms, respectively.

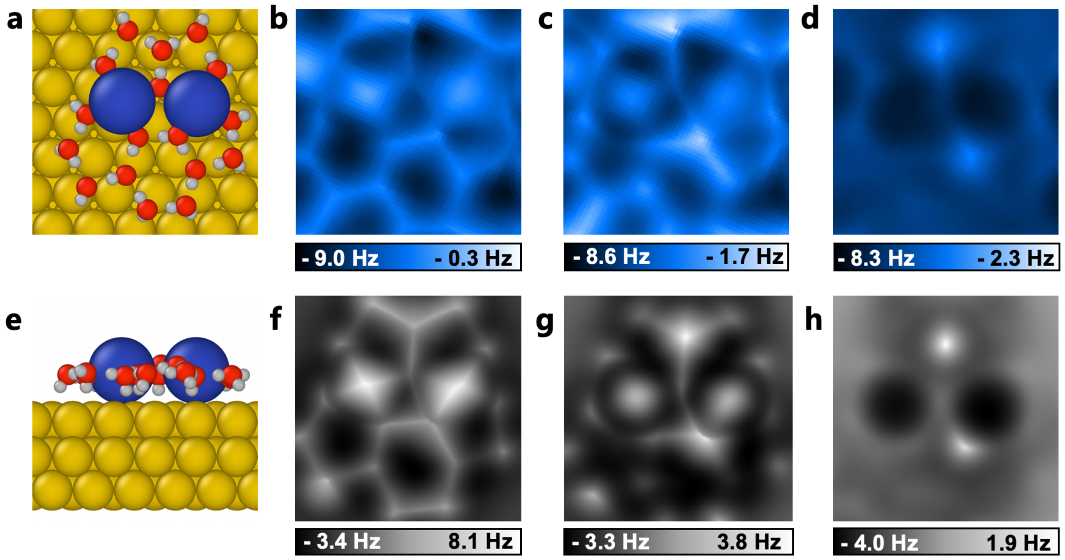

Figure S11 | Validation of the predicted K^+ hydrates structure. (a and e), Atomic model (a: top view; e: side view) according to the NN prediction of experimental data in fig. S10. The red, white, indigo and golden spheres represent O, H, K and Au, respectively. (b-d), Experimental AFM images of the K^+ hydrates at different tip heights of -235 pm (b), -205 pm (c) and -145 pm (d). (f-h), Simulated AFM images of the K^+ hydrates at increasing tip heights from left to right. The tip heights in b-d are referenced to the STM set point on the K^+ cation (100 mV, 10 pA). The tip heights in f-h are defined as the vertical distance between the apex atom of the metal tip and the outmost atom of Au substrate. The oscillation amplitude of experimental and simulated images is 100 pm.

15 The prediction performance of NN with or without transfer learning

We compare the prediction performance of NN with or without transfer learning as the training dataset size varies. The same validation dataset is used regardless of how much training data there is and whether or not the NN training uses transfer learning techniques. As shown in fig. S12, we chose the structures on which the NN without transfer learning performs best according to MSE, however, the NN trained without transfer learning still cannot predict the position of hydrogen atoms and the water molecule orientation.

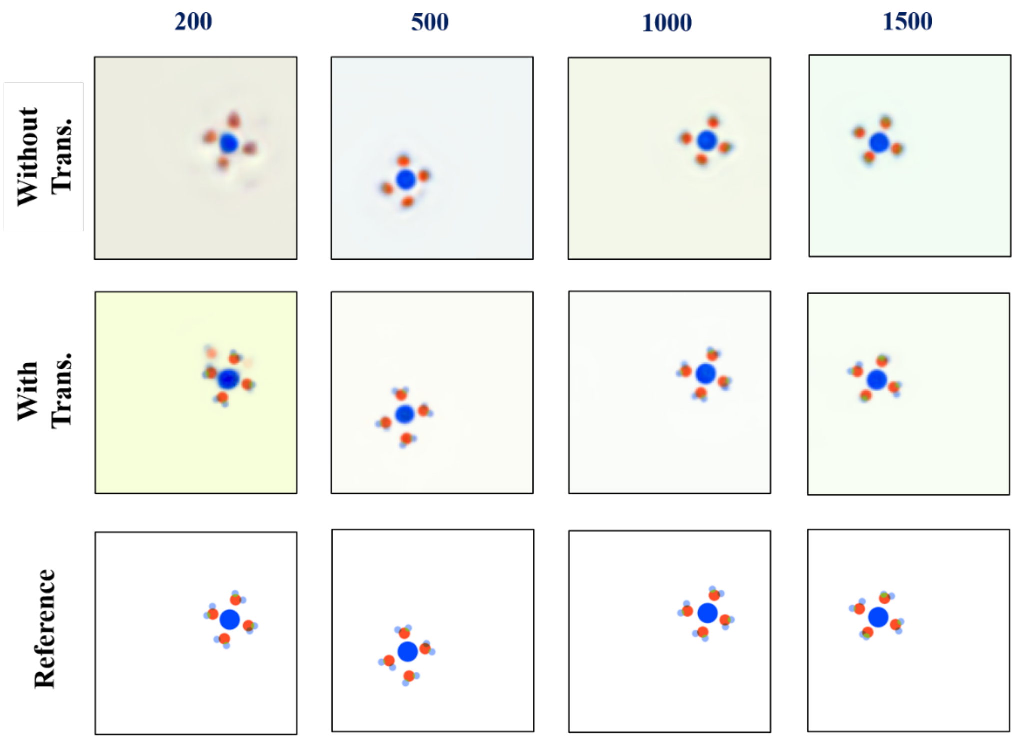

Figure S12 | Prediction of Na hydrates by NN with or without transfer learning. The first and second rows are the representation of the $Na^{+}\cdot 4H_2O$ predicted by NN with or without transfer learning, respectively; the third row is the reference representation. The number of training data of NN varies from left to right and is denoted at the top of each column. For each column, the structure on which the NN without transfer learning performs best according to MSE is chosen.

16 Object detection for each atom

Negative and positive charge maps are used for atom detection. For the detection of oxygen atoms and sodium ions, we take pixel grayscale and pixel number as the criteria for atom detection and identification. For each element, any pixel with grayscale g_p satisfying $g_p \in [g^i_{min}, g^i_{max}]$ can be considered as part of it. After finding all relevant pixels, we consider all connected pixels as one possible atom and count their number N. If the number of those pixels satisfies $N \in [N^i_{min}, N^i_{max}]$, this block of pixels can be regarded as an atom j representing element i. To obtain the coordinates of each detected atom j, we average the coordinates of the pixels belonging to it and set the average to $\boldsymbol{r}_j$. For each atom j, we calculate its distance from the corresponding atom

in the label. Once the distance is less than the threshold d_{match}, we consider it as a true positive (TP) prediction. Missing and mismatched predicted atoms are treated as false negative (FN) and false positive (FP) prediction, respectively. Since the atoms are detected one by one, to avoid one detected atom affecting the recognition of other atoms, we use a circle mask of radius r to cover the detected atoms.

For the hydrogen atoms, specifically, only a few pixels of light grayscale are presented. We use the edge detection for atom identification. The image is fist smoothed by operator S_s

$$S_s = \begin{pmatrix} 0 & 1 & 0 \\ 1 & 6 & 1 \\ 0 & 1 & 0 \end{pmatrix} \qquad \text{(S6)}$$

Then, we use 2 sober operators, S_x and S_y, to compute the gradients on the x-axis and y-axis, resulting in scalar gradients at each position. Where the gradients falling into the range $[gr_{min}, gr_{max}]$ can be considered as edges. The pixels of the region bounded by each connected edge should also be within the range $[N^H_{min}, N^H_{max}]$. Furthermore, we should consider the shape of the region as an important criterion. We define the shape factor of a region as follows:

$$f = \sum_i R_i^2 / (\sum_i R_i)^2, \qquad \text{(S7)}$$

where R_i denotes the distance from the i-th pixel to the center of the region, and the sum is over all pixel at the edge of the region. A perfect circle has a shape factor of 1. Once the $f > f_{max}$ where f_{max} is a threshold, the shadow should be discarded. In addition, if two hydrogen atoms overlap in the label and we can only detect one region in the prediction, we count them as two TPs.

Finally, for each element, the prediction accuracy is calculated by eq. S7 based on all predictions for it in the target dataset.

$$accuracy = \frac{TP}{TP+FP+FN}. \qquad \text{(S8)}$$

To avoid overestimating the performance of the NN, the parameter set in the prediction accuracy calculation algorithm was adjusted and cross-checked by manual calculated prediction accuracy of each atom species. We manually identified the atoms and

calculate the prediction accuracy for each atom species among different datasets. Calculated using the same datasets used in Fig. 4, we plot the error bars with the manually collected data (see fig. S13). The prediction accuracy of machine calculation of this parameter set is slightly lower than that of manual calculation, indicating that the prediction accuracy of the machine calculation is reliable.

Table S4 Parameters used in atom detection

Element	H	O	Na/K
g_{min}		25	0
g_{max}		50	75
N_{min}	9	3	10
N_{max}	50	100	200
gr_{min}	23		
gr_{max}	96		
d_{match}[Å]	0.2	0.2	0.4
r[Å]	0.6	0.8	1.6
f_{max}	1.15		

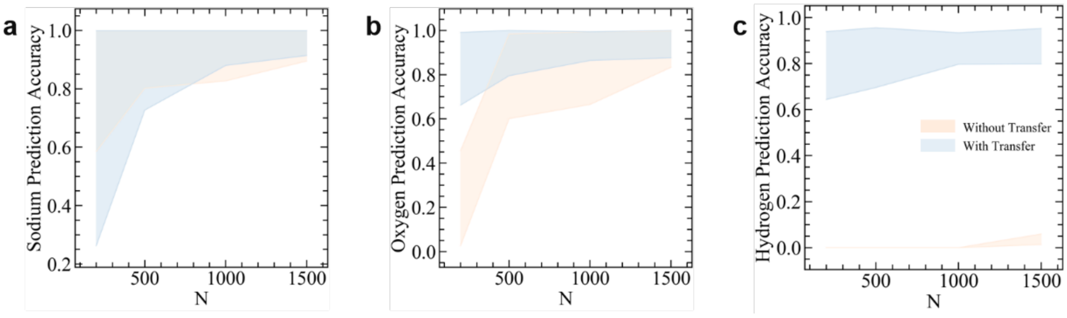

Figure S13 | The prediction accuracy error bar in Fig. 4 with the manually collecting data. a, **b** and **c** are the positional accuracies of sodium, oxygen and hydrogen atoms, respectively. The blue and orange color patched represent the training process with and without pretrained parameter loading, respectively. The upper and lower bounds of the color patches are manually calculated from the data at the top and bottom 20% of the loss ranking, respectively.

Reference

1. Çiçek, Ö, Abdulkadir, A, Lienkamp, SS*, et al.* 3D U-Net: learning dense volumetric segmentation from sparse annotation. In: *International conference on medical image computing and computer-assisted intervention, 2016*, p. 424-32. Springer.
2. Litjens, G, Kooi, T, Bejnordi, BE*, et al.* A survey on deep learning in medical image analysis. *Medical image analysis*. 2017; **42**: 60-88.
3. Plimpton, S. Fast parallel algorithms for short-range molecular dynamics. *Journal of computational physics*. 1995; **117**(1): 1-19.
4. AMBER 14;University of California, San Francisco, 2014.
5. Fuentes-Azcatl, R, Barbosa, MC. Sodium chloride, NaCl/epsilon: new force field. *J Phys Chem B*. 2016; **120**(9): 2460-70.
6. Leontyev, IV, Stuchebrukhov, AA. Polarizable molecular interactions in condensed phase and their equivalent nonpolarizable models. *J Chem Phys*. 2014; **141**(1): 014103.
7. Yagasaki, T, Matsumoto, M, Tanaka, H. Lennard-Jones parameters determined to reproduce the solubility of NaCl and KCl in SPC/E, TIP3P, and TIP4P/2005 water. *Journal of Chemical Theory and Computation*. 2020; **16**(4): 2460-73.
8. Peng, JB, Cao, DY, He, ZL*, et al.* The effect of hydration number on the interfacial transport of sodium ions. *Nature*. 2018; **557**(7707): 701-5.
9. Ryckaert, JP, Ciccotti, G, Berendsen, HJC. Numerical integration of the cartesian equations of motion of a system with constraints: molecular dynamics of n-alkanes. *Journal Of Computational Physics*. 1977; **23**(3): 327-41.
10. Perdew, JP, Burke, K, Ernzerhof, M. Generalized gradient approximation made simple. *Phys Rev Lett*. 1996; **77**(18): 3865.
11. Hamann, D. H 2 O hydrogen bonding in density-functional theory. *Phys Rev B*. 1997; **55**(16): R10157.
12. Kresse, G, Furthmüller, J. Efficient iterative schemes for ab initio total-energy calculations using a plane-wave basis set. *Phys Rev B*. 1996; **54**(16): 11169.
13. Klimeš, J, Bowler, DR, Michaelides, A. Chemical accuracy for the van der Waals density functional. *Journal of Physics: Condensed Matter*. 2009; **22**(2): 022201.
14. Klimeš, J, Bowler, DR, Michaelides, A. Van der Waals density functionals applied to solids. *Phys Rev B*. 2011; **83**(19): 195131.
15. Jorgensen, WL, Tirado-Rives, J. The OPLS [optimized potentials for liquid simulations] potential functions for proteins, energy minimizations for crystals of cyclic peptides and crambin. *Journal of the American Chemical Society*. 1988; **110**(6): 1657-66.
16. Stukowski, A. Visualization and analysis of atomistic simulation data with OVITO–the Open Visualization Tool. *Modelling and simulation in materials science and engineering*. 2009; **18**(1): 015012.
17. Kingma, DP, Ba, J. Adam: A method for stochastic optimization. *arXiv preprint arXiv:14126980*. 2014.
